# COVID-19 Outbreak in an Acute-Care Hospital: Lessons Learned

**DOI:** 10.1017/ash.2021.109

**Published:** 2021-07-29

**Authors:** Supriya Narasimhan, Vidya Mony, Tracey Stoll, Sherilyn Oribello, Karanas Yvonne, Dolly Goel

## Abstract

**Background:** We describe the infection prevention investigation of a cluster of 15 healthcare workers (HCWs) and 7 patients in a single non–COVID-19 unit of an acute-care hospital in September 2020. **Methods:** The infection prevention team was notified of 13 SARS-CoV-2–positive, symptomatic HCWs in an acute-care non–COVID-19 unit in 1 week (August 30, 2020, to September 3, 2020). In the same week, 2 patients who had been on the unit were diagnosed with nosocomial COVID-19. An epidemiologic investigation identified the exposure period to be between August 19, 2020, and September 3, 2020. The following immediate containment measures were implemented: closing the unit to new admissions, restricting float staff, moving existing patients to private rooms, mandatory masking of patients, and mandatory respirator and eye protection on unit entry for all HCWs. Exposed unit staff were tested immediately and then every 4 days until September 18, 2020. Likewise, exposed patients, including those discharged, were notified and offered testing. Hospital-wide HCW surveillance testing was conducted. Enhanced environmental control measures were conducted, including terminal cleaning and ultraviolet C (UV-C) disinfection of common areas and patient rooms and a thorough investigation of airflow. Detailed staff interviews were performed to identify causes of transmission. Multiple town hall meetings were held for staff education and updates. **Results:** In total, 108 total patients were deemed exposed: 33 were inpatients and 75 had been discharged. Testing identified 5 additional patient cases among 57 patients who received testing; 51 chose to self-monitor for symptoms. Staff testing identified 2 additional cases. Thus, 15 HCWs and 7 patients were linked in this cluster. The containment measures successfully ended staff transmission as of September 5, 2020. The last patient case was detected on September 10, 2020. Secondary cases were noted in 6 HCW families. We identified staff presenteeism, complacency, and socialization in break rooms and outside work as major causes of transmission. Suboptimal compliance with universal eye protection and hand hygiene (67%) were contributing factors. We determined by contact tracing and temporality that the outbreak could have stemmed from nursing home patient(s) through floating HCWs to staff on the affected unit. Directionality of transmission was from staff to patients in this cluster. **Conclusions:** Many facets of pandemic fatigue were apparent in this outbreak, namely, inability of HCWs to adhere to changing PPE guidance, presenteeism pressures due to workforce needs, and socialization with peers due to a false sense of security conferred by biweekly surveillance testing. Ongoing PPE education, repeated reinforcement, as well as engagement in staff wellness are crucial to combatting pandemic fatigue, conserving our workforce, and preventing future outbreaks.

**Funding:** No

**Disclosures:** None

